# Woody Plant Diversity and Community Structure Along Elevational and Soil Gradients in *Betula platyphylla* Forests, Southeastern Tibetan Plateau

**DOI:** 10.1002/ece3.73707

**Published:** 2026-05-27

**Authors:** Ngawang Norbu, Rong‐fang Wang, Dorgon Dolma, Sumdurp Tashi, Yixi Jiacuo, Zhe‐fei Zeng, Ngawang Bonjor, Norzin Tso, Jun‐wei Wang, La Qiong

**Affiliations:** ^1^ Key Laboratory of Biodiversity and Environment on the Qinghai‐Tibetan Plateau, Ministry of Education, School of Ecology and Environment Xizang University Lhasa China; ^2^ Yani Observation and Research Station for Wetland Ecosystem of the Xizang Autonomous Region Xizang University Nyingchi China

**Keywords:** *Betula platyphylla* forest, elevation, environmental factors, plant community, spatial distribution, species diversity

## Abstract

As a climate‐sensitive region on a global scale, how environmental gradients on the Tibetan Plateau influence species diversity and community structure is an important scientific issue for understanding community assembly mechanisms in alpine ecosystems. This study focused on *Betula platyphylla* forests in the Nyang River Basin in the southeastern Tibetan Plateau (elevation 2990–4100 m), where a total of 16 plots (48 quadrats of 10 × 10 m) were established, field surveys were conducted, and hierarchical cluster analysis, canonical correspondence analysis (CCA), regression analysis, and Pearson correlation analysis were applied. The aims were to (1) explore the response patterns of woody plant diversity in *B. platyphylla* forests along the elevational gradient; and (2) evaluate the effects of key environmental factors, including elevation and soil properties, on the community distribution patterns of *B. platyphylla* forests. The results showed that a total of 71 woody plant species were recorded, belonging to 22 families and 40 genera, mainly represented by Rosaceae, Ericaceae, and Caprifoliaceae. It can be classified into four associations (Ass): *B. platyphylla* + *Rhododendron bulu*, *B. platyphylla* + *Dasiphora fruticosa* , *Quercus aquifolioides* + *Lonicera tangutica*, and *B. platyphylla* + *Rhododendron triflorum*. With increasing elevation, the species Margalef richness index (*M*), Shannon‐Wiener diversity index (*H*), Patrick richness index (*Pa*), and Simpson diversity index (*D*) showed a significant decline (*p* < 0.05), while the Simpson dominance index (*C*) increased significantly (*p* < 0.05). The Pielou evenness index (*J*) did not significantly respond to elevational gradients (*p* > 0.05). CCA showed that environmental factors jointly explained 37.80% of the variation in community composition, among which elevation (Elev), soil alkali‐hydrolyzable nitrogen (AN), organic matter (OM), and available phosphorus (AP) were the main environmental factors affecting the woody plant community composition of *B. platyphylla* forests (*p* < 0.05). This study provides key baseline data on plant diversity of *B. platyphylla* forests in this region and offers a scientific basis for the conservation and management of alpine forest ecosystems under climate change.

## Graphical abstract

This study investigates the species composition, distribution, and diversity of *Betula platyphylla* forests in the Nyang River Basin in southeastern Tibet. The results show that elevation is the primary factor influencing species diversity in plant communities, with diversity indices significantly decreasing with increasing elevation. The study also found that environmental factors such as soil alkali nitrogen, organic matter, and available phosphorus have a significant impact on plant community distribution, providing scientific support for the conservation of mountain forest plant diversity in the Tibetan Plateau.

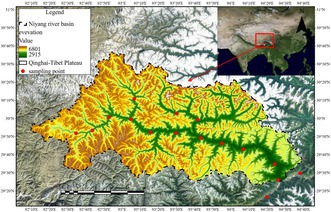

## 1 Introduction

Understanding how plant community composition and species diversity vary along environmental gradients is a core issue in vegetation ecology and biogeography (Zhang et al. 2022), reflecting species' resource use strategies and their ecological roles within communities, and providing insights into ecosystem structure, function, and responses to global change (Zheng et al. 2024). These patterns serve as the theoretical foundation for understanding ecosystem structure, function, and their responses to global changes (Dietrich et al. 2024; Zhang et al. 2022). The elevational gradient, due to its ability to comprehensively reflect the systematic changes in multiple ecological factors such as temperature, precipitation, soil development, and resource availability over relatively short spatial scales (Sundqvist et al. 2013), is considered a “natural laboratory” for studying the relationship between biodiversity and the environment (Rahbek et al. 2019). Studies have shown that the diversity of species with different life forms along environmental gradients varies significantly, with woody plants exhibiting more distinct distribution patterns compared to herbaceous plants (O'Brien 1993). Therefore, studying the diversity of woody plants along elevational gradients is of significant importance and value for revealing species distribution patterns and mechanisms.

Forest ecosystems, as an essential component of terrestrial ecosystems, play a crucial role in maintaining global biodiversity, regulating climate, conserving water, and sequestering carbon, among other ecosystem services (Anandita et al. 2024). Under the influence of global climate change, the structure and function of forest ecosystems are undergoing significant alterations (Huang et al. 2025), particularly in mountainous areas where environmental conditions change rapidly over small spatial scales (Storms et al. 2025). Numerous studies have revealed various patterns in the change of species α‐diversity along elevational gradients: species diversity may increase monotonically with elevation (Cui and Zheng 2016), decrease monotonically (Unger et al. 2010), or follow a unimodal distribution (Dani et al. 2023; He et al. 2024). These patterns are often attributed to factors such as environmental filtering, resource availability, and habitat heterogeneity, with the specific form of these patterns being highly dependent on the study subjects, spatial scale, and regional ecological context. Studies suggest that high‐elevation forest communities are typically dominated by a few cold‐tolerant, nutrient‐poor species. As elevation increases, forest communities generally undergo a shift from species‐rich, structurally complex communities to communities with fewer species and more prominent dominant species (Rawal et al. 2025; Wang, Liu, et al. 2025). However, the response of different forest types and dominant species to elevational gradients shows significant regional variation (Sharma et al. 2017).

Soil, as one of the most important environmental factors in forest ecosystems, serves as a critical link between vegetation and the environment (Bradley et al. 2025). The physicochemical properties of soil directly influence plant nutrient acquisition, growth strategies, and interspecies competition, and shape community species composition and diversity patterns across different spatial scales (Bell et al. 2000; Li et al. 2025). During changes along environmental gradients, soil development, nutrient accumulation, and distribution patterns often undergo significant alterations with climate conditions and vegetation types, thus becoming key factors driving changes in plant diversity (Brambach et al. 2017; Unger et al. 2010).

Studies have shown that soil processes in alpine and arid regions strongly influence hydrological dynamics, nutrient availability, and ecosystem resilience (Wu et al. 2023). In high‐elevation ecosystems, soil moisture, structure, and organic matter jointly regulate plant growth and carbon cycling (Wu et al. 2025). Soil properties reflect underlying biogeochemical processes that control resource availability and ultimately affect plant growth (Zaman et al. 2025). Therefore, understanding these interactions is crucial for elucidating community assembly mechanisms and maintaining ecosystem stability under climate change.

The Tibetan Plateau is one of the most climate‐sensitive regions globally (Wu, Liu, et al. 2024; Wu, Hu, et al. 2024), and is widely regarded as a hotspot for studying ecosystem responses to climate change (Zhang, Zou, et al. 2023; Zhang et al. 2023a). The Nyang River Basin, located in the southeastern Tibetan Plateau, is home to rich plant diversity, with significant vertical differentiation in vegetation types. These include alpine shrub meadows, subalpine coniferous forests, and mixed broadleaf‐conifer forests. Common tree species in the region include *Betula platyphylla* , *Picea likiangensis* var. *linzhiensis*, *Quercus aquifolioides*, and *Rhododendron bulu* (Wang et al. 2023). The *B. platyphylla* forests, widely distributed in the basin, are located in the harsh environments of the mid‐ to high‐elevation zones. They are not only typical representatives of alpine forests but also crucial components of regional forest succession and ecological stability. However, how the community structure and species diversity of woody plants in *B. platyphylla* forests respond to elevation and soil factors (OM, AN, AP, and pH) remains unclear. Therefore, this study focused on *B. platyphylla* forests in the Nyang River Basin to systematically analyze the effects of elevation and soil factors on community composition and species diversity. This study not only provides key baseline data on plant diversity of *B. platyphylla* forests in this region, but also establishes a scientific foundation for the conservation and management of alpine forest ecosystems under climate change, offering important reference for biodiversity monitoring and management in high‐altitude ecosystems.

## 2 Materials and Methods

### 2.1 Study Area Overview

The study area is located in the Nyang River Basin in the southeastern Tibetan Plateau (E92°10′–94°35′, N29°30′–30°30′). It is the second‐largest tributary of the Yarlung Tsangpo River, with elevation ranging from 2935 m to 5030 m, displaying distinct alpine canyon topography. The terrain gradually decreases from west to east, with significant vegetation zonation and rich biodiversity (Wang et al. 2023). The region is influenced by the combined effects of warm, humid air from the Indian Ocean and cold air masses from the north, resulting in a typical plateau temperate monsoon climate. The average annual temperature is approximately 8.5°C, with an annual precipitation of 842 mm. The annual runoff primarily occurs from June to September, and evaporation can reach up to 1766 mm (Hao et al. 2022; Zou et al. 2025). As one of the major tributaries of the Yarlung Tsangpo River, the Nyang River Basin plays a crucial role in regional water conservation, soil preservation, and ecological security. Its forest vegetation is essential for maintaining the ecological functions and biodiversity of the basin.

### 2.2 Plot Setup and Field Survey

**FIGURE 1:**
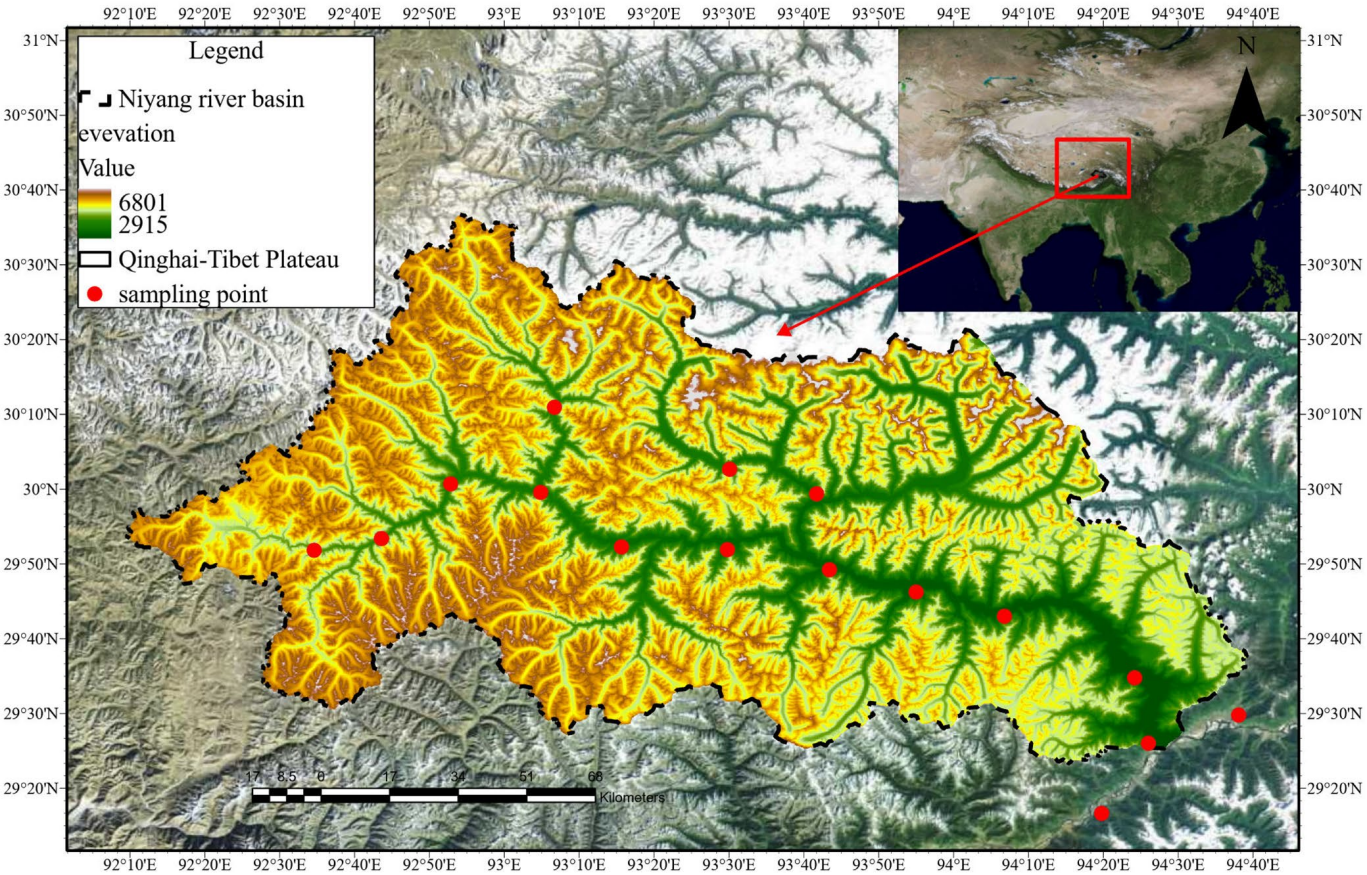
Geographical location and distribution of sampling plots in the Nyang River basin.

From July to August 2024, a survey of woody plant diversity was conducted in *B. platyphylla* forests. To cover the full elevational range of *B. platyphylla* distribution (2990–4100 m) in the Nyang River Basin and to capture spatial heterogeneity, 16 plots were established, with three 10 m × 10 m subplots in each plot spaced more than 50 m apart. A total of 48 *B. platyphylla* forest subplots were surveyed for vegetation (Figure 1). Surface soil samples (0–20 cm) from each subplot were collected using a five‐point sampling method and combined into mixed samples. Detailed records of plant species composition, individual counts, average height, and average cover were documented for each subplot. The geographical coordinates, including elevation, latitude, and longitude, of each subplot were recorded using a Garmin eTREX handheld GPS device.

### 2.3 Data Processing and Analysis

#### 2.3.1 Diversity Measures

This study used the Margalef richness index (*M*), Simpson diversity index (*D*), Shannon–Wiener diversity index (*H*), Patrick richness index (*Pa*), Simpson dominance index (*C*), and Pielou evenness index (*J*) to comprehensively characterize species richness, evenness, and dominance of woody plant communities in *B. platyphylla* forests. The formulas for calculation are presented in Table 1 (Li et al. 2023, 2025).

**TABLE 1:** Formulas for calculating species importance value (IV) and species diversity indices.

Species IV and diversity index	Calculation formulas	Calculation formulas
Species importance value	$\mathrm{IV}=\left(\mathrm{Ar}+\mathrm{Hr}+\mathrm{Cr}\right)/3$	*Ar* ‘relative abundance’ = (number of plants of a particular species)/(total number of plants of all species) × 100% *Cr* ‘relative coverage’ = (sum of coverage of all individuals of a particular species)/(total coverage of all species) × 100% *Hr* ‘relative height’ = (sum of the heights of all individuals of a particular species)/(total height of all species) × 100%
Margalef richness index	$M=\frac{S-1}{\ln N}$	*S* is the number of species in the quadrat, *N* is the sum of the numbers of all species. *P*_*i*_ = *N*_*i*_/*N*, *P*_*i*_ is the proportion of the *i* th species, and *N*_*i*_ is the number of individuals of the *i* th species.
Simpson diversity index	$D=1-\sum_{i=1}^{S}\;P_{i}^{2}$	
Shannon–Wiener diversity index	$H=-\sum_{i=1}^{S}\;P_{i}\ln P_{i}$	
Patrick richness index	Pa=S	
Simpson dominance index	$C=\sum_{i=1}^{S}\;p_{i}^{2}$	
Pielou evenness index	J=H/lnS	

#### 2.3.2 Measurement of Soil Physicochemical Properties

The physicochemical properties of the soil were measured using standard methods (Liu et al. 2024): Soil pH was determined using the glass electrode method, which reflects soil acidity and alkalinity and influences nutrient availability and microbial activity, thereby regulating plant nutrient uptake; total nitrogen (TN) was measured using the Kjeldahl method, and total phosphorus (TP) and available phosphorus (AP) were measured using the molybdenum–antimony colorimetric method; total potassium (TK) and available potassium (AK) were determined using the flame photometric method. TN, TP, and TK represent the total nutrient pool, while AN, AP, and AK indicate the availability of nutrients for immediate plant uptake. Organic matter (OM) was measured using the potassium dichromate hot oxidation method, serving as an important indicator of soil fertility, nutrient storage, and water‐holding capacity. Alkali‐hydrolyzable nitrogen (AN) content was determined using the alkali‐hydrolyzable diffusion method, and soil dry matter content (SDMC) was measured using the gravimetric method, reflecting soil moisture status and structural stability.

#### 2.3.3 Data Analysis

In this study, species importance values were used as the measurement basis, hierarchical cluster analysis was performed using Ward's method, and plant community types in the study area were identified based on the Euclidean distance. Linear regression analysis was applied to examine the variation trend of community species diversity along the elevational gradient, and Pearson correlation analysis was used to test the relationships between species diversity indices and environmental factors. These analyses were conducted using the Vegan and Stats packages in R version 4.3.3 (R Core Team 2023). Canonical Correspondence Analysis (CCA) was employed to quantify the regulatory effects of environmental gradients on community composition, and this analysis was performed in Canoco 5 software (Braak and Smilauer 2012). The significance level for all statistical tests in this study was set at *p* < 0.05.

## 3 Results and Analysis

### 3.1 Species Composition and Quantitative Classification of *Betula platyphylla* Forest Community

A total of 71 woody plant species were surveyed in the *B. platyphylla* forest community, belonging to 22 families and 40 genera. In terms of family composition, the most species‐rich families were Rosaceae (20 genera, 28.1%), Ericaceae (8 genera, 11.2%), Caprifoliaceae (7 genera, 9.8%), Salicaceae (6 genera, 8.4%), and Pinaceae (4 genera, 5.6%). In terms of genus composition, the most abundant genera were *Rhododendron* (7 species, 9.8%), *Lonicera* (7 species, 9.8%), *Rosa* (4 species, 5.6%), *Cotoneaster* (4 species, 5.6%), and *Salix* (4 species, 5.6%). The dominant species in the basin included *B. platyphylla* , *Q. aquifolioides*, *R. bulu*, *Berberis gyalaica*, and *Lonicera tangutica*, reflecting their high tolerance to low temperatures and nutrient‐poor soils at high elevations, as well as their efficient nutrient acquisition strategies, which enable them to outcompete other species and become dominant under these environmental constraints (Table 2).

**TABLE 2:** Main dominant species of the *Betula platyphylla* forest community.

Species name	Family	Genus	Importance value (Mean ± SD)
*Betula platyphylla*	Betulaceae	*Betula*	27.43 ± 13.62
*Quercus aquifolioides*	Fagaceae	*Quercus*	11.42 ± 13.72
*Rhododendron bulu*	Ericaceae	*Rhododendron*	5.17 ± 7.90
*Berberis gyalaica*	Berberidaceae	*Berberis*	4.02 ± 4.64
*Lonicera tangutica*	Caprifoliaceae	*Lonicera*	3.52 ± 3.68
*Salix daltoniana*	Salicaceae	*Salix*	3.23 ± 5.74
*Cotoneaster submultiflorus*	Rosaceae	*Cotoneaster*	3.18 ± 4.47
*Populus rotundifolia* var. *duclouxiana*	Salicaceae	*Populus*	2.85 ± 6.22
*Dasiphora fruticosa*	Rosaceae	*Dasiphora*	2.56 ± 4.81
*Lyonia villosa*	Ericaceae	*Lyonia*	2.50 ± 4.15
*Picea likiangensis* var. *linzhiensis*	Pinaceae	*Picea*	2.28 ± 4.36
*Rhododendron triflorum*	Ericaceae	*Rhododendron*	2.23 ± 4.18
*Rhododendron vellereum*	Ericaceae	*Rhododendron*	1.87 ± 5.88
*Cotoneaster acutifolius*	Rosaceae	*Cotoneaster*	1.70 ± 2.67
*Rosa sericea*	Rosaceae	*Rosa*	1.69 ± 3.51
*Pinus densata*	Pinaceae	*Pinus*	1.53 ± 3.64
*Rhododendron lulangense*	Ericaceae	*Rhododendron*	1.40 ± 4.99

**FIGURE 2:**
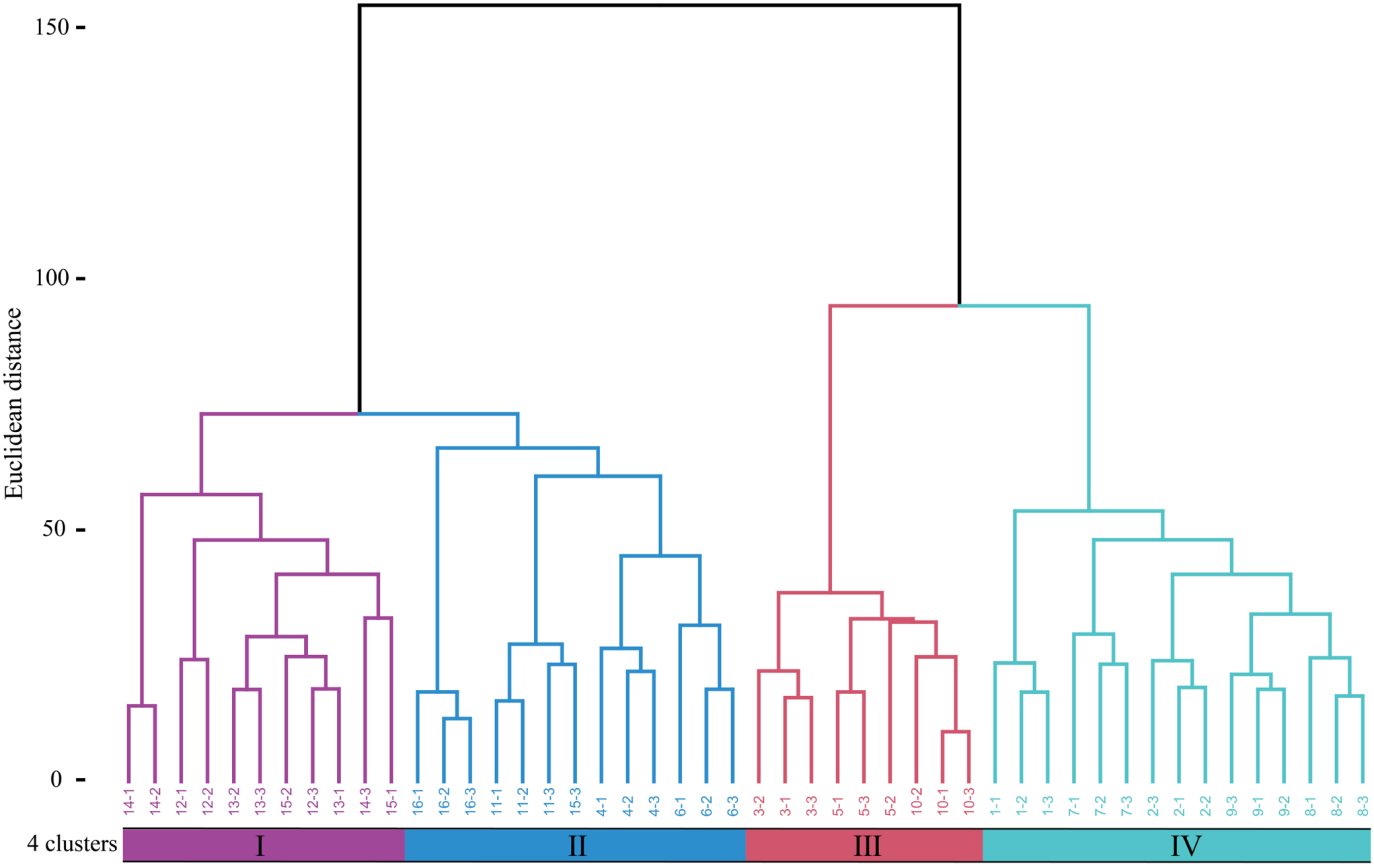
Ward's hierarchical cluster dendrogram of *Betula platyphylla* forest plant community. I–IV: Association types, the numbers represent the plot numbers.

Ward's cluster analysis was conducted on 48 community plots of the *B. platyphylla* forest. On the basis of plant community classification and naming principles (Lai et al. 2010), the 48 community plots were divided into 4 associations (Ass), with the following Ass names (Figure 2):

Ass I: *B. platyphylla* —*R. bulu* Ass includes plots 12, 13, 14, 15–1, and 15–2, totaling 11 subplots. The dominant tree species in the tree layer is *B. platyphylla* , with accompanying species such as *Salix daltoniana*, *Populus rotundifolia* var. *duclouxiana*, and *Rhododendron vellereum*. The dominant species in the shrub layer is *R. bulu*, with accompanying species including *L. tangutica*, *Cotoneaster submultiflorus*, and *Dasiphora fruticosa* .

Ass II: *B. platyphylla* — *D. fruticosa* Ass, includes plots 4, 6, 11, 15–3, and 16, totaling 13 subplots. The dominant tree species in the tree layer is *B. platyphylla* , with accompanying species such as *Q. aquifolioides*, *P. likiangensis* var. *linzhiensis*, and *Rhamnus virgata*. The dominant species in the shrub layer is *D. fruticosa* , with accompanying species including *R. bulu*, *B. gyalaica*, and *Rosa sericea* .

Ass III: *Q. aquifolioides*—*L. tangutica* Ass, includes plots 3, 5, and 10, totaling 9 subplots. The dominant tree species in the tree layer is *Q. aquifolioides*, with accompanying species such as *B. platyphylla* , *P. rotundifolia* var. *duclouxiana*, and *Sorbus rehderiana*. The dominant species in the shrub layer is *L. tangutica*, with accompanying species including *Lyonia villosa*, *Cotoneaster acutifolius* , and *C. submultiflorus*.

Ass IV: *B. platyphylla* —*Rhododendron triflorum* Ass includes plots 1, 2, 7, 8, and 9, totaling 15 subplots. The dominant tree species in the tree layer is *B. platyphylla* , with accompanying species such as *Q. aquifolioides*, *P. likiangensis* var. *linzhiensis*, and *Abies georgei*. The dominant species in the shrub layer is *R. triflorum* , with accompanying species including *L. villosa* , *C. submultiflorus*, and *B. gyalaica*.

### 3.2 Elevational Gradient Distribution Pattern of *B. platyphylla* Forest Community Diversity

**FIGURE 3:**
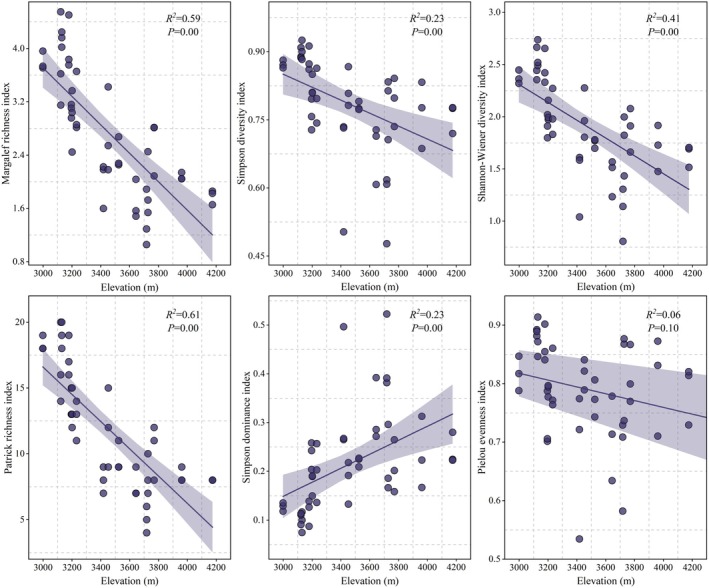
Variation pattern of species diversity index in *Betula platyphylla* forest along the elevational gradient. The trend line and shaded area represent the fitted values of the linear regression and its 95% confidence interval, respectively.

As shown in Figure 3, the *M* ranged from 1.06 to 4.55, with a mean value of 2.72; the *D* ranged from 0.48 to 0.93, with a mean value of 0.78; the *H* ranged from 0.81 to 2.74, with a mean value of 1.91; the *Pa* ranged from 4 to 20, with a mean value of 11.77; the *C* ranged from 0.07 to 0.52, with a mean value of 0.22; and the *J* ranged from 0.53 to 0.91, with a mean value of 0.79.

Overall, the species diversity distribution of woody plants in the Nyang River Basin shows significant variation along the elevational gradient. The *M* (*R* ^2^ = 0.59, *p* = 0.00), *D* (*R* ^2^ = 0.23, *p* = 0.00), *H* (*R* ^2^ = 0.41, *p* = 0.00), and *Pa* (*R* ^2^ = 0.61, *p* = 0.00) all exhibited consistent trends along the elevational gradient, with a significant decreasing trend as elevation increased. In contrast, the *C* (*R* ^2^ = 0.23, *p* = 0.00) showed a significant increasing trend with elevation. The *J* (*R* ^2^ = 0.06, *p* = 0.10) showed no significant change with elevation. This pattern may result from environmental filtering: at high elevations, only cold‐tolerant and nutrient‐efficient species (such as *R. bulu*, *D. fruticosa* , and *Rhododendron kongboense*) can survive, thereby reducing overall species richness while increasing the relative abundance of these dominant species. Overall, the woody plant diversity in *B. platyphylla* forests of the Nyang River Basin is at a moderately high level, with an average of 12 species per plot, and some plots reaching up to 20 species.

### 3.3 Relationship Between Plant Community Distribution Patterns and Environmental Factors

Through CCA analysis of 48 community plots and 10 environmental factors in the study area, the results showed that the explanatory power of the environmental factors reached 37.80%. The Monte Carlo permutation test for the relationship between environmental factors and community distribution yielded an *F* value of 2.0 and a *p* value of 0.002. Therefore, the CCA results can, to some extent, effectively explain the relationship between plant community distribution and environmental factors.

**FIGURE 4:**
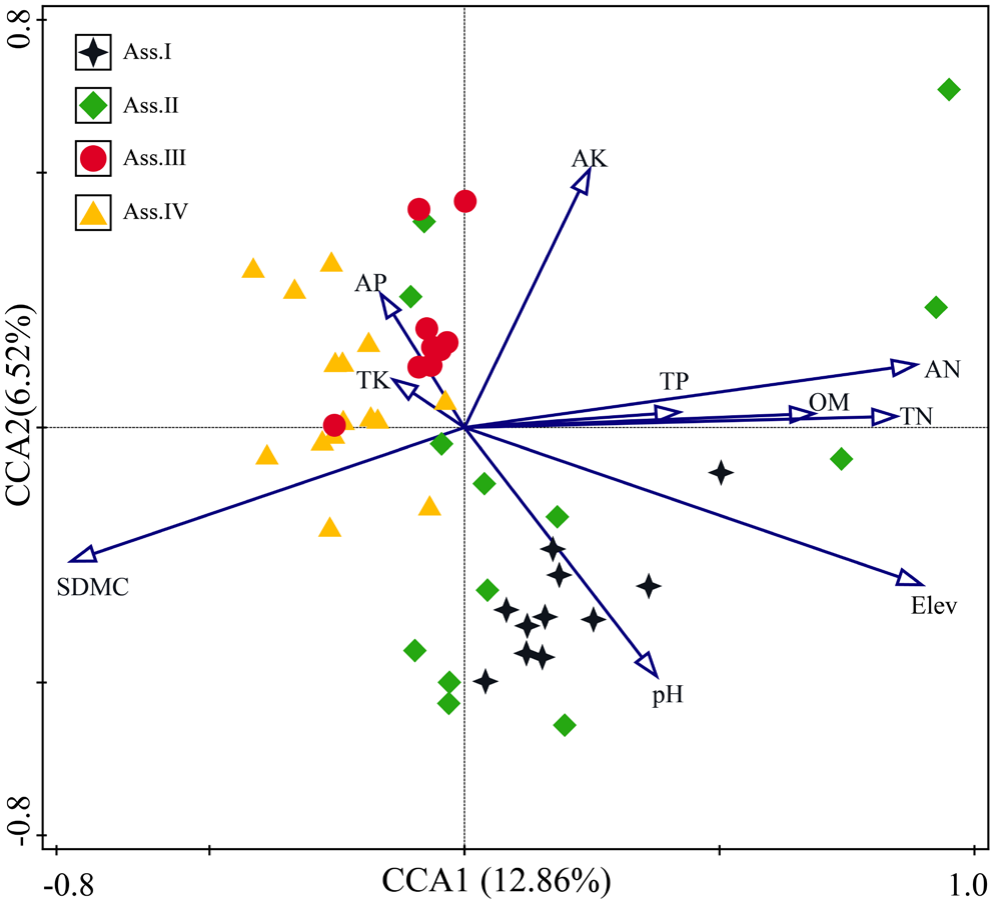
CCA of plant community distribution and environmental factors. Soil pH (pH), soil total nitrogen (TN), soil total phosphorus (TP), soil available phosphorus (AP), soil total potassium (TK), soil available potassium (AK), soil organic matter (OM), soil alkali‐hydrolyzable nitrogen (AN), soil dry matter content (SDMC), elevation (Elev).

As shown in Table 3 and Figure 4, the first two axes of the CCA reflect the combined effects of multiple environmental factors. Among these, Elev has the highest contribution at 30%, followed by soil AN, AP, and pH. The environmental factors that determine the first ordination axis are, in order, Elev, AN, OM, SDMC, TN, and TP. The first axis is significantly positively correlated with Elev, AN, and OM, indicating that the first ordination axis mainly reflects changes in plant community distribution along the gradients of Elev, AN, and OM. The primary environmental factors determining the second ordination axis are AP, AK, and pH. The second axis is significantly positively correlated with AP and significantly negatively correlated with pH, indicating that the second ordination axis reflects changes in plant community distribution along the gradients of AP and pH. On the basis of the CCA ordination diagram and environmental factor significance tests, it can be concluded that Elev, AN, OM, AP, and pH have the greatest influence on community distribution and are the dominant factors determining the spatial distribution of plant communities.

**TABLE 3:** Results of Monte Carlo permutation tests for environmental variables in CCA.

Environmental variables	Contribution %	Pseudo‐*F*	*p*
Elev	30	5.4	0.002
AN	12.8	2.4	0.002
AP	10.2	1.9	0.008
OM	9.3	1.8	0.012
pH	8.6	1.7	0.018
SDMC	7.5	1.5	0.062
TK	7.5	1.5	0.054
TP	5.6	1.1	0.282
TN	5.1	1	0.444
AK	3.3	0.6	0.932

*Note:* Soil pH (pH), soil total nitrogen (TN), soil total phosphorus (TP), soil available phosphorus (AP), soil total potassium (TK), soil available potassium (AK), soil organic matter (OM), soil alkali‐hydrolyzable nitrogen (AN), soil dry matter content (SDMC), elevation (Elev).

Based on the results of Ward's cluster analysis and CCA ordination, the distribution patterns of the four associations can be observed (Figure 2, Figure 4). The distribution patterns of community types along the elevational gradient show significant differentiation, with environmental gradient changes driving the gradual variation in community species composition. Ass I and Ass II are primarily distributed in high‐elevation areas with higher AN, OM, and pH. In contrast, Ass III and Ass IV are mainly distributed in areas with higher AP content, lower elevation, and lower AN content. These distribution differences may be closely related to the regulatory effects of soil nutrients: higher levels of soil AN and OM may promote the growth of dominant species by providing essential nutrients, thereby enhancing their competitive advantage and shaping community structure along the elevational gradient.

### 3.4 Relationship Between Species Diversity and Environmental Factors

**FIGURE 5:**
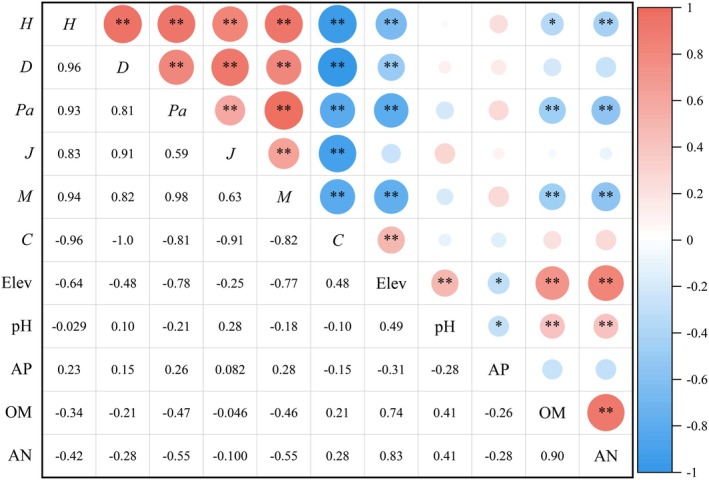
Pearson correlation analysis between species diversity indices and environmental factors. Shannon‐Wiener diversity index (*H*), Simpson diversity index (*D*), Patrick richness index (*Pa*), Pielou evenness index (*J*), Margalef richness index (*M*), Simpson dominance index (*C*), elevation (Elev), soil pH (pH), soil available phosphorus (AP), soil organic matter (OM), soil alkali‐hydrolyzable nitrogen (AN).

Pearson correlation analysis was conducted between the species diversity indices and the five environmental factors that most significantly influenced plant community distribution according to the CCA ordination, including Elev, AN, OM, AP, and soil pH. The results revealed varying degrees of correlation between the species diversity indices and environmental factors (Figure 5).

Elev is significantly negatively correlated with the *H*, *D*, *Pa*, and *M* (*p* < 0.01), while it is significantly positively correlated with the C (*p* < 0.01). OM and AN are significantly negatively correlated with *H*, *Pa*, and *M* (*p* < 0.05), indicating that higher soil nutrient levels favor the growth of a few dominant species, which outcompete subordinate species, leading to a decline in community species richness and shaping community structure through environmental filtering and dominance effects. The *J* showed no significant correlation with any of the environmental factors. These findings suggest that the key factors influencing the species diversity of *B. platyphylla* forests in the Nyang River Basin are Elev, AN, and OM, while AP and pH have no significant effect on community species diversity (*p* > 0.05).

## 4 Discussion

### 4.1 Classification of Plant Communities and Environmental Explanation of Their Distribution

The classification of plant communities is an important method for studying community composition and spatial distribution patterns (Cáceres and Wiser 2012). Common classification methods include cluster analysis, two‐way indicator species analysis (TWINSPAN), and multivariate regression trees (MRT) (Zhang, Zou, et al. 2023a, 2023b). Among these, cluster analysis calculates the similarity distance between plots and groups similar plots into one class to define community types. CCA analysis can effectively reveal the degree of correlation between environmental factors and community distribution patterns, thus providing a more intuitive ecological explanation of the complex relationship between communities and environmental factors (ter Braak 1986).

Based on the results of Ward's cluster analysis, this study shows that the plant communities of *B. platyphylla* forests in the study area can be divided into four main association types along the elevational gradient: *B. platyphylla* —*R. bulu* Association (Ass I), *B. platyphylla* — *D. fruticosa* Association (Ass II), *Q. aquifolioides*—*L. tangutica* Association (Ass III), and *B. platyphylla* — *R. triflorum* Association (Ass IV). Among these, Ass III and Ass IV are mainly distributed in relatively low or mid‐elevation areas, where the communities have richer accompanying species and more complex community structures. In these areas, species such as *Q. aquifolioides* and *L. tangutica* dominate, adapting to the fertile, warm, and humid low‐elevation environments. The higher levels of AP and lower levels of AN in the low‐elevation areas may provide sufficient nutrient support for these species, thereby promoting their growth and expansion. In contrast, Ass I and Ass II are primarily distributed in higher elevation areas, where the community structure tends to be simplified, with a concentration of ecological niches of dominant species. These species exhibit strong cold resistance and drought tolerance, allowing them to adapt to the cold, nutrient‐poor high‐elevation environment. Topography is considered an important factor influencing species composition and distribution (Lan et al. 2011). The CCA ordination results indicate that elevation is the primary environmental factor affecting the distribution of *B. platyphylla* forest communities. He et al. (2024) found that elevation is the dominant factor influencing plant community distribution patterns in mountain ecosystems; Worthy et al. (2019) also demonstrated that elevation significantly affects plant community distribution patterns in the Andes Mountains. This community replacement pattern along the elevational gradient is consistent with the vertical zonation observed in alpine forest ecosystems.

The CCA analysis also revealed that soil AN, AP, OM, and pH have a significant impact on community distribution (*p* < 0.05, Figure 4, Table 3). This result indicates that the spatial pattern of *B. platyphylla* forest communities is the result of the combined effects of the elevational gradient and soil nutrient gradients. Among these, elevation changes influence plant community structure in mountain forests and are a decisive factor in limiting species distribution and community type (Wang, Chen, et al. 2025). As the elevational gradient changes, ecological factors such as temperature, humidity, sunlight, and soil undergo synergistic changes. The combined effects of these environmental factors, through the mechanism of environmental filtering, select plant species, thereby shaping the spatial distribution patterns of plant communities (Chawla et al. 2008; Rahman, Afzal, et al. 2021; Rahman, Khan, et al. 2021). This study confirms that elevation has a significant impact on the distribution pattern of *B. platyphylla* forest communities. Therefore, elevation is an important environmental factor influencing the spatial distribution of *B. platyphylla* forest communities in the Nyang River Basin.

Soil is the material basis for plant growth, and its spatial heterogeneity leads to differences in resource utilization among plants, which in turn determines the spatial distribution patterns of plant communities (Xue et al. 2024). This study indicates that elevation and key soil factors (OM, AN, pH, AP) play important roles in regulating the community structure of *B. platyphylla* forests. Low temperatures at high elevations slow the decomposition of soil organic matter, promoting OM accumulation and enhancing nitrogen storage (Meng et al. 2024; Rotich et al. 2025), thereby providing favorable conditions for the growth and resource use of dominant species. Soil pH regulates nutrient mineralization and influences plant‐available nutrients (Wang and Kuzyakov 2024; Khan et al. 2019), which facilitates the establishment and dominance of nutrient‐conservative, stress‐tolerant species (Ohdo and Takahashi 2020). Soil AP, as a readily absorbed nutrient, directly determines plant phosphorus acquisition (Vance et al. 2003; Wu et al. 2022); in high‐elevation areas, low temperatures and strong fixation limit AP availability, further influencing community composition. This species replacement and distribution differentiation along the nutrient gradient ultimately lead to significant distribution pattern differences in plant communities across different elevational zones (I. U. Rahman, Afzal, et al. 2021; Rahman, Khan, et al. 2021; Vincent et al. 2014; Albornoz et al. 2021). Research by Birhanu et al. (2021) indicated that elevation, soil OM, pH, and P are the main environmental factors driving species differences in African mountain forest communities. Similarly, Mumshad et al. (2021) found that soil pH, OM, and elevation are significant factors influencing species distribution and plant community formation, which aligns with the conclusions of this study.

Overall, these results indicate that elevational gradients act as strong environmental filters by imposing climatic constraints, while soil nutrients (such as OM, AN, and AP) regulate community assembly through biogeochemical processes. Specifically, higher levels of soil OM and AN may enhance the resource acquisition capacity of dominant species, intensifying competitive exclusion and thereby reducing species coexistence. This suggests that plant community assembly in *B. platyphylla* forests is jointly regulated by environmental filtering and resource limitation mechanisms, which are fundamental processes shaping alpine plant communities.

### 4.2 Vertical Distribution Characteristics of Plant Community Diversity and Its Relationship with Soil Factors

Species composition forms the foundation of plant communities and is one of the most fundamental characteristics of plant communities (Sadia et al. 2024). In this study, a total of 71 woody plant species belonging to 22 families and 40 genera were found in the *B. platyphylla* forests of the Nyang River Basin at elevations of 2990–4100 m. The dominant families were Rosaceae, Ericaceae, and Caprifoliaceae. Species diversity includes both richness and evenness (Zhang 2018). In recent years, ecological research has gradually shifted from describing single diversity indices to a multidimensional, multi‐indicator analysis of species diversity patterns and their ecological mechanisms (Yan et al. 2023). This shift reflects the species richness, evenness, and dominance characteristics of communities from different perspectives.

In this study, species diversity follows a monotonically decreasing distribution pattern with increasing elevation. The *M*, *D*, *H*, and *Pa* all significantly decrease with elevation, while the *C* significantly increases. This pattern is consistent with findings from other regions, such as the Himalayas (Bhat et al. 2020) and the Andes Mountains (Worthy et al. 2019), regarding the species diversity of woody plants along elevational gradients. Similarly, Li (2022) also found a significant negative correlation between the species diversity of woody plants and elevation in the Changbai Mountains of China. In this study, the *J* showed no significant change along the elevational gradient, but overall, the *J* index was relatively high. Higher evenness is beneficial for maintaining community structure stability (Hillebrand et al. 2008). This study also found that along the elevational gradient, community species diversity indices increased in parallel with species richness, exhibiting a consistent trend, which aligns with studies in the northwestern Himalayas (Wani et al. 2022).

Species diversity patterns along the elevational gradient are closely related to environmental factors. Among these factors, the soil environment is widely recognized as a major constraint on plant diversity and directly influences community composition and distribution (Sun et al. 2023). Although many studies have explored the interactions between species diversity and soil factors, the results remain highly variable among different regions and ecosystems (Tiawoun et al. 2022; Veríssimo et al. 2024). In this study, Pearson correlation analysis showed that soil OM and AN have significant effects on species diversity.

Soil OM and AN are significantly negatively correlated with *H*, *Pa*, and *M*, but show no significant correlation with *C* or *J*. This pattern is consistent with the results reported by Yang et al. (2016) in primary forests of Hainan Island and by Hao et al. (2022) in studies on *Alternanthera philoxeroides* . Increasing levels of OM and AN impose strong environmental filtering effects, which preferentially select species with higher nutrient acquisition and utilization capacities. This selective advantage promotes rapid biomass accumulation and the formation of dominance by these species, thereby intensifying competitive exclusion and limiting the establishment of coexisting species (Gough et al. 2000), as a result, community species diversity and richness are reduced.

Recent studies conducted in the southeastern Tibetan Plateau and adjacent high‐elevation regions have revealed patterns similar to those observed in this study. Previous research has shown that elevational gradients and soil nutrient conditions can significantly influence species diversity and composition of alpine plant communities, among which soil OM and AN are often regarded as key regulating factors (Han et al. 2022; Zhang et al. 2025), consistent with our findings. Existing studies further indicate that elevation not only acts as an environmental filter through climatic constraints such as low temperature, but also indirectly affects community assembly by influencing soil nutrient accumulation and transformation. In particular, soil OM and AN are not only soil factors closely related to plant growth status, but also dynamic components of the carbon–nitrogen cycle (Chen et al. 2024; Sokol et al. 2022). OM contributes to soil carbon storage and provides substrates for microbial decomposition, thereby promoting nutrient release, while AN represents the pool of plant‐available nitrogen that continuously cycles through mineralization, microbial uptake, and plant assimilation. Therefore, variations in OM and AN along the elevational gradient can regulate nutrient availability and resource fluxes, influence interspecific competition, and ultimately shape community composition and assembly processes.

Studies have shown that in high‐elevation mountainous areas, processes such as debris flows, landslides, and intense convective weather can alter soil development and habitat heterogeneity, affecting the spatial distribution of resources and thereby indirectly influencing species composition and community dynamics (Kulakowski et al. 2017; Yang et al. 2026). Therefore, it is necessary in future studies to integrate hydrological processes, geomorphic disturbances, and long‐term monitoring to further assess their potential impacts on the structure and stability of *B. platyphylla* forest ecosystems.

## 5 Conclusions

This study systematically investigated the composition, structure, elevational distribution patterns of species diversity, and driving factors of woody plant communities in *B. platyphylla* forests of the Nyang River Basin. The main conclusions are as follows:

1. A total of 71 woody plant species were recorded, belonging to 22 families and 40 genera, with Rosaceae, Ericaceae, and Caprifoliaceae as the dominant families. The dominant tree species were *B. platyphylla* , *Q. aquifolioides*, and *P. likiangensis* var. *linzhiensis*, while the dominant shrub species were *R. bulu*, *D. fruticosa* , and *L. tangutica*.
2. With increasing elevation, Margalef richness index (*M*), Simpson diversity index (D), Shannon–Wiener diversity index (H), and Patrick richness index (*Pa*) showed significant decreases, whereas Simpson dominance index (*C*) increased significantly. Pielou evenness index (*J*) showed no significant change. These results indicate that community structure at higher elevations is increasingly dominated by a few species. Pearson correlation analysis further revealed that elevation, soil OM, and AN are the key environmental factors driving changes in species diversity patterns.
3. Along the elevational gradient, *B. platyphylla* forests can be classified into four typical association types: *B. platyphylla—R. bulu* Association (Ass I), *B. platyphylla—D. fruticosa* Association (Ass II), *Q. aquifolioides—L. tangutica* Association (Ass III), and *B. platyphylla—R. triflorum* Association (Ass IV). Elev, soil AN, AP, OM, and soil pH jointly shape the spatial distribution patterns of plant communities.

The results of this study provide important guidance for alpine ecosystem security and management. By identifying key environmental drivers, such as elevation and soil nutrients, priority areas for conservation and restoration can be determined: mid‐elevation areas with high soil fertility may serve as refugia for biodiversity, while high‐elevation areas require management strategies to alleviate environmental stress and enhance ecosystem resilience. Elevation and soil factors (AN, OM, AP, and pH) jointly shape the community structure and species diversity of *B. platyphylla* forests through environmental filtering and resource limitation mechanisms. These processes favor the survival of a few stress‐tolerant dominant species while limiting overall species richness, thereby driving the spatial assembly of communities along elevational and environmental gradients. Understanding these mechanisms provides a scientific basis for biodiversity conservation and adaptive forest management of high‐altitude *B. platyphylla* forests under climate change.

## Author Contributions

**Ngawang Norbu:** investigation (equal), methodology (equal), software (equal), visualization (equal), writing – original draft (equal). **Rong‐fang Wang:** investigation (equal), methodology (equal), writing – original draft (equal). **Dorgon Dolma:** investigation (equal), methodology (equal), writing – original draft (equal). **Sumdurp Tashi:** methodology (equal), software (equal), writing – original draft (equal). **Yixi Jiacuo:** methodology (equal), software (equal), writing – original draft (equal). **Zhe‐fei Zeng:** investigation (equal), methodology (equal), writing – original draft (equal). **Ngawang Bonjor:** methodology (equal), software (equal). **Norzin Tso:** methodology (equal), software (equal). **Jun‐wei Wang:** conceptualization (equal), investigation (equal), methodology (equal), writing – review and editing (equal). **La Qiong:** conceptualization (equal), funding acquisition (equal), methodology (equal), writing – review and editing (equal).

## Funding

This work was supported by Xizang Autonomous Region Science and Technology Program, XZ202402ZD0005, XZ202402JX0003, XZ202402ZY0023, XZ202401ZR0028, XZ202502JD0005. Xizang University Graduate High‐Level Talent Training Program, 2025‐GSP‐S092. Monitoring and Operation Project of the Forestry Ecological Station, National Forestry and Grassland Administration, 2025132077.

## Conflicts of Interest

The authors declare no conflicts of interest.

## Supporting information


**Data S1:** ece373707‐sup‐0001‐DataS1.zip.

## Data Availability

The data that supports the findings of this study are available in the Supporting Information of this article.
